# Exhaust the exhausters: Targeting regulatory T cells in the tumor microenvironment

**DOI:** 10.3389/fimmu.2022.940052

**Published:** 2022-09-30

**Authors:** Bayley R. McRitchie, Billur Akkaya

**Affiliations:** ^1^ Department of Neurology, The College of Medicine, The Ohio State University, Columbus, OH, United States; ^2^ Pelotonia Institute for Immuno-Oncology, The James Comprehensive Cancer Center, The Ohio State University, Columbus, OH, United States; ^3^ Department of Microbial Infection and Immunity, The College of Medicine, The Ohio State University, Columbus, OH, United States

**Keywords:** tumor, T cell exhaustion, immunotherapy, regulatory T cell, Treg, mechanism

## Abstract

The concept of cancer immunotherapy has gained immense momentum over the recent years. The advancements in checkpoint blockade have led to a notable progress in treating a plethora of cancer types. However, these approaches also appear to have stalled due to factors such as individuals’ genetic make-up, resistant tumor sub-types and immune related adverse events (irAE). While the major focus of immunotherapies has largely been alleviating the cell-intrinsic defects of CD8^+^ T cells in the tumor microenvironment (TME), amending the relationship between tumor specific CD4^+^ T cells and CD8^+^ T cells has started driving attention as well. A major roadblock to improve the cross-talk between CD4^+^ T cells and CD8^+^ T cells is the immune suppressive action of tumor infiltrating T regulatory (Treg) cells. Despite their indispensable in protecting tissues against autoimmune threats, Tregs have also been under scrutiny for helping tumors thrive. This review addresses how Tregs establish themselves at the TME and suppress anti-tumor immunity. Particularly, we delve into factors that promote Treg migration into tumor tissue and discuss the unique cellular and humoral composition of TME that aids survival, differentiation and function of intratumoral Tregs. Furthermore, we summarize the potential suppression mechanisms used by intratumoral Tregs and discuss ways to target those to ultimately guide new immunotherapies.

## Introduction

**Figure 1:**
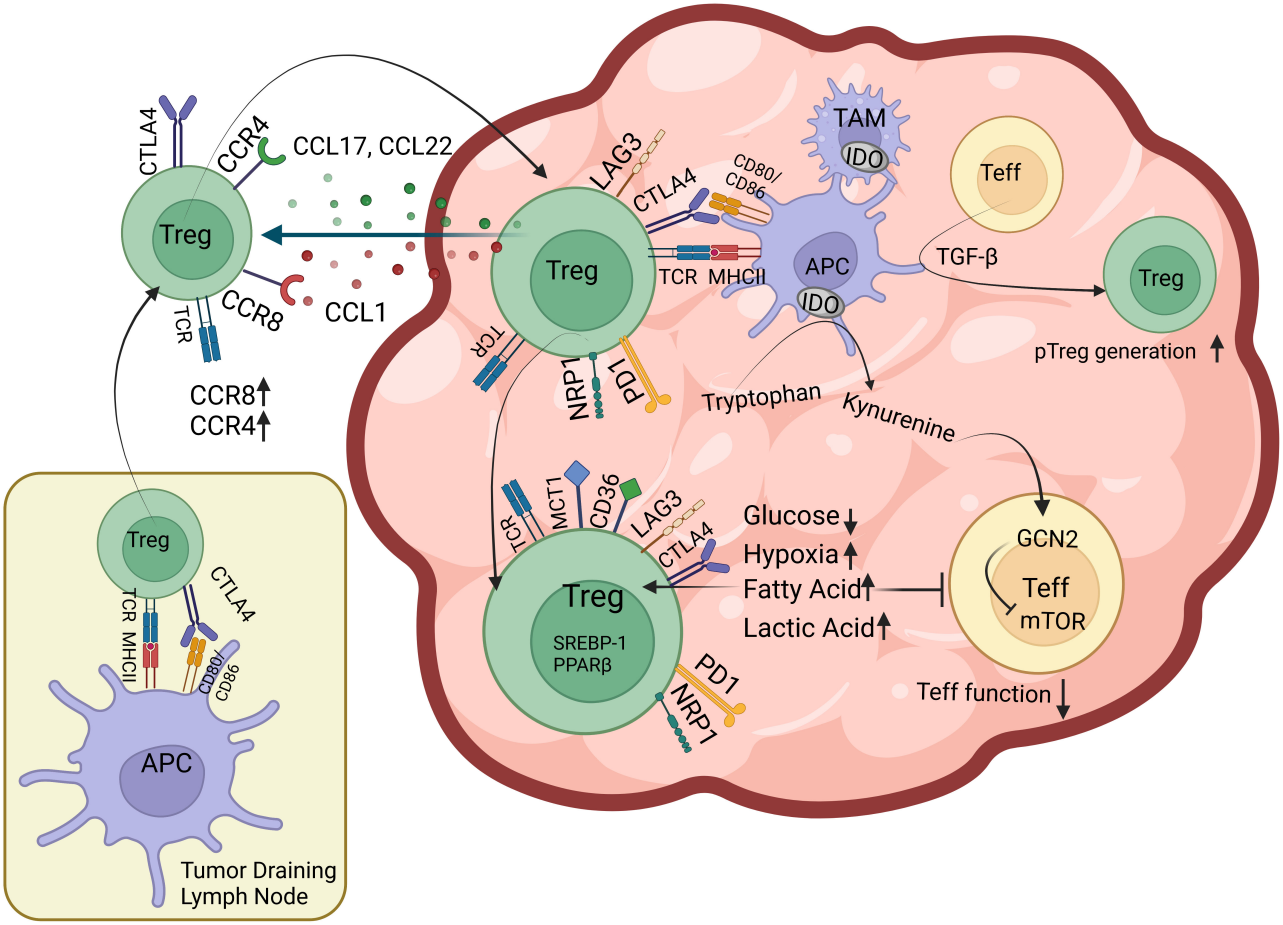
Factors that facilitate Treg infiltration of the TME Tregs that are presented tumor antigens by professional APCs in the tumor draining lymph node upregulate chemokine receptors including CCR4 and CCR8 and migrate towards CCL17, CCL22, CCL1 gradient created by TME. Once in tumor tissue, Tregs have subsequent encounters with intratumoral APCs where they upregulate surface expression of coinhibitory receptors including PD-1 and LAG3. Intratumoral Tregs also express CTLA-4, Nrp-1, lactic acid transporter MCT-1 and free fatty acid (FFA) scavenging receptor CD36 on their surface as well as nuclear factors such as PPAR family of transcription factors and SREBP-1 responsible for FFA oxidation. TGF-β from tumor cells, stroma and immune cells induce intratumoral pTreg generation. Tumor and immune cell IDO activity and reduced glucose concentration of TME inhibits mTOR in effector T cells (Teff) and inhibit their function.

Tregs constitute a small yet significant subset of CD4^+^ T cells with a distinct immune suppressive role (1). Their primary function is enforcing peripheral tolerance and is largely dictated by the activity of transcription factor Foxp3. Treg ontogeny involves thymic Tregs (tTregs), that develop in the thymus from precursors of CD4^+^ helper T (Th) cells and peripheral Tregs (pTregs), that differentiate from mature CD4^+^ Th cells in the periphery. A third Treg type is known as induced Treg (iTregs) that can be produced ex-vivo from mature CD4^+^ Th cells by providing T cell receptor (TCR) stimulus and TGF-β (2). A dual expression of Foxp3 and IL-2 receptor α chain (CD25) accompanied by a low IL-7 receptor (CD127) expression is widely accepted as the hallmark of Treg ontogeny in mammals (3). Suppression mechanisms of Tregs can be grouped into active and counteractive modes, where active mode represents the production of immune suppressive cytokines by Tregs such as IL-10, TGF-β, IL-35, adenosine and counteractive mode includes removal of crucial elements for the activation and survival of effector T cells such as peptide-MHC class II, CD80-CD86, and IL-2 (4–12) (**Figure 1**). While it is largely unknown which mechanism(s) predominate *in vivo*, it is widely accepted that stimulation of Treg TCR precedes the suppressive activity.

The major role played by Tregs is to prevent autoimmunity as evidenced by the fatal multiorgan autoimmunity in patients with IPEX (Immune dysregulation, Polyendocrinopathy, Enteropathy, X-linked) syndrome and *Scurfy* mice that harbor deleterious Foxp3 mutations (13, 14). Despite critical importance, Treg activity is not desired in certain niches, such as sites of chronic infection and the tumor microenvironment (TME). It has been shown that the changes associated with tumor growth, such as altered nutrient composition and oxygen availability, cytokines and chemokines released by tumor cells, stroma and immune cells also favor Treg infiltration and effector T cell exhaustion. These also is a correlation with disease progression and resistance to immune checkpoint inhibitors (ICI), hence Treg infiltration of the TME has started to be appreciated as a biomarker and predictive factor for tumor progression and therapy response (15, 16). Moreover, this highlights the need for more specific approaches to reduce Treg to effector T cell ratio in the TME. To help achieve this goal, this review focuses on the factors that recruit Tregs to the tumor site, promote their differentiation and activity in the TME as well as biomarkers that distinguish tumor-specific Tregs from tumor-specific CD4^+^ effector T cells and self-specific Tregs that protect against autoimmunity.

## How do Tregs populate the tumor microenvironment?

### Antigen-T cell receptor interactions

TCR and antigen-MHC interactions constitute the essence of T cell development, function, and tolerance. T cell precursors start to recognize antigens in thymic cortex as soon as they successfully assemble a pre-TCR complex on their membrane. Pre-TCRs that are capable of binding self pMHC, get through positive selection, continue rearranging TCRα loci in both alleles, then enter thymic medulla and encounter another set of self pMHCs. This encounter subjects fully rearranged TCRαβ chains to a negative selection, which then determines the T cell fate. Although it is still debated, traditionally it has been assumed that the affinity of a single interaction between individual TCR molecule and self pMHC determines the fate of T cells precursors. In this affinity-based model for negative selection, the strongest signal leads to elimination of the T cell clone altogether, weak signals lead to generation of conventional (effector) T cells while the signals in between tend to produce Tregs (1, 17–19). However, more recent studies support an avidity-based model where the fate of CD4^+^ T cell precursor is based on the timing and density of the interactions between TCR and self-antigens as the major determinant (20–22). This allows for T cells that have fewer reactions with antigens to become conventional T cells, T cells that have the highest number of interactions with self-antigens to be deleted, and the T cells that have moderate amounts of interaction with self-antigen to become Tregs (20). A common feature for both models, is that they demonstrate the TCR-pMHCII interactions as the key determining factor for T cell fate. However, it’s also possible that costimulatory pathways, integrins, and cytokines further tune the TCR signals and ultimately contribute to the lineage decision for Tregs vs. effector T cells (23–25).

Antigens presented at the TME have been heavily investigated due to their potential use as cancer vaccines (26). These antigens can be divided into two large groups according to their expression in normal tissues. Tumor associated antigens (TAAs) can be expressed in normal tissues hence, the high affinity T cell clones specific for TAAs are mostly deleted from the repertoire during thymic development. Therefore, their use for immunotherapies is limited due to the lack of good effectors that would use them and the possibility of off-target toxicity against normal tissues. Because TAA-specific Tregs that are present in the repertoire unlike effector T cells, it is possible TAA presentation can selectively recruit them. The second group of antigens is tumor specific antigens (TSA), also known as neoantigens. These occur due to mutations in tumor genome, thus perceived as foreign by immune cells. They can induce significant anti-tumor T cell response as TCR repertoire can display a full spectrum of affinities for foreign antigens. However, their role in Treg recruitment and conversion is largely unknown (27–29).

TCR-pMHCII interactions are also critical for mature Tregs to home and function in the periphery. Because mature Treg repertoire is equipped to detect self pMHCII, a pathological expression of tissue antigens on MHCII could possibly engage and expand particular Treg clones in the secondary lymphoid organs (30, 31). Depending on the inflammatory context that tissue-specific Tregs see their antigen, they can unleash a myriad of suppressive mechanisms such as the removal of self pMHCII and CD80-86 from APC surface, IL-2 from potential autoimmune clonal escapees and an active production of anti-inflammatory mediators (4–12, 32). Tumor site gradually diverges from adjacent healthy tissue due to increasing mutational burden, emerging tumor stroma, and new blood vessels. Hence, antigenic profile of tumor deviates from normal tissue where those antigens can be presented on MHCI and MHCII by both tumor itself and the APCs within tumor and draining lymph node (33). This potentially helps tumor-reactive bona fide tTregs to migrate into tumor site. An alternative way tumor site can be enriched in Tregs is the differentiation of tumor-reactive effector CD4^+^ T cells into pTregs. This path is mostly attributed to the role of TGF-β in inducing Foxp3 expression in effector CD4^+^ T cells in addition to its prominent role in transforming the architectural and immune scapes of TME (34–39)

Tregs display a limited TCR repertoire within the TME, suggestive of a clonal enrichment for Tregs that recognize TAAs and TSAs (40, 41). Due to a lack of reliable markers that distinguish the origin of Tregs, it is largely unclear to what extent pTregs vs tTregs make up these clones (42). Based on limited overlap between the TCR repertoires of tumor infiltrating effector CD4^+^ T cells and Tregs, some have argued that pTregs do not play a prominent role in the TME (15, 43, 44). However, a single snapshot of the TIL repertoire may be misleading due to a number of factors including differences in the metabolic adaptation and survival of Tregs and effector T cells in TME. It is possible that TME imposes a bottle-neck for the incoming tumor-specific CD4^+^ T cells due to hostile metabolic environment where the ones that differentiate into pTregs survive and others face their demise. This may gradually build a tumor-specific Treg repertoire composed of metabolically adapted tTregs and pTregs, while CD4^+^ effector T cell repertoire may reflect a constantly replenished pool from periphery. Hence a single repertoire analysis can potentially capture relatively older Treg clones together with freshly migrating CD4^+^ effector T cells reacting to a changing antigen scape. Moreover, as tumor progresses, clonal representation of effector T cells may be actively skewed by Tregs *via* antigen-specific suppression mechanisms. We have previously shown that antigen-specific Tregs can steal their cognate pMHCII from DCs, resulting in an antigen-biased suppression *in vivo* (45). If this is a prominent suppression mechanism in the TME, it may be another reason why some effector CD4^+^ and CD8^+^ T cell clones vanish from TIL repertoire while others remain (46, 47).

### Migration and homing of Tregs to tumor site

Tregs that are primed by dendritic cells in secondary lymphoid organs upregulate an array of chemokine receptors and adhesion molecules can then migrate into tissue (48, 49). CCR4 and CCR8 expression on Tregs have long been implicated in Treg recruitment to TME (39, 50–53). CCR4 responds to CCL17 and CCL22, mostly released from several immunologically active “hot” tumors, while CCR8 responds to CCL1 gradient (51, 54, 55). It has been shown that inhibition of CCR4 signaling diminishes Treg infiltration of the TME while Treg numbers are maintained in the periphery (54). Furthermore, depleting CCR4^+^ Tregs seems to reduce the tumor burden both as a standalone therapy and in combination with checkpoint blockade in certain tumor settings (54, 56–60). Despite the potential of targeting CCR4 for immunotherapies, exact role of CCR4 in Treg development, function and tumor-Treg crosstalk remains elusive (61). To properly address these points, future studies using constitutive and inducible conditional knockout animal models, where CCR4 deficiency is restricted to Tregs, are required. Similar to CCR4, CCR8 has also been noted for its specificity to Treg that have infiltrated the TME (39, 62). While antibody mediated depletion of CCR8^+^ Tregs *via* antibody dependent cell cytotoxicity (ADCC) can ameliorate the tumor burden, blocking CCR8 activity using antibodies that are devoid of ADCC capabilities doesn’t seem to create the same effect (52). Furthermore, comparison of CCR8 knockout and wild type mice revealed that Tregs infiltrate the TME similarly, suggesting a trivial role for CCR8 in Treg recruitment and function (63). Similar to CCR4, the contribution of CCR8 to Treg recruitment needs to be further elucidated in mouse models that specifically lacks CCR8 in Tregs. Hypoxic environment of TME has also been implicated in recruitment of Tregs along with myeloid derived stromal cells (MDSCs) and tumor-associated macrophages (TAMs) (64–66). Hypoxia driven release of CCL28 from tumors has been shown to recruit CCR10 expressing Tregs that contribute to angiogenesis by VEGF production (67–69). Tregs also require cell adhesion molecules including ICAM-1, L-selectin, P-selectin, integrin αE (CD103) and VLA-4 to home to secondary lymphoid organs and inflammation site (70). While majority of these factors also play role in effector T cells homing, Nrp-1-Semaphorin-4a axis has recently been recognized for its unique role in enriching a stable, suppressive intratumoral Treg population in mice and human (71–74). Although Nrp-1 is also known as a co-receptor for vascular endothelial growth factor (VEGF) that regulates endothelial cell adhesion and angiogenesis, to what extent it plays role in Treg homing to TME remains to be elucidated (75).

### Metabolism

Changing nutrient, metabolite and oxygen composition of the tumor affect presence and function of anti- and pro-tumor immune cells (76–78). Upon activation, naïve T cells switch from oxidative phosphorylation (OXPHOS) to aerobic glycolysis to meet the needs of activated T cells (79). This metabolic adaptation is similar to the Warburg effect seen in cancer cells, where tumor prefers quick yet less efficient energy supply from glycolysis over OXPHOS regardless of oxygen tension (80). However, not only this depletes glucose in TME and puts effector T cells in a hyporesponsive state, but also hinders T cell wellbeing via increasing lactate (80, 81). While effector T cells suffer in this hostile landscape, Tregs thrive largely due to their adaptations for utilizing fatty acids and lactate (82–87) (**Figure 1**). T cell activation in the presence of CD28 stimulation upregulates machinery for glycolysis *via* PI3K/Akt/mTOR (the mammalian target of rapamycin) pathway. mTOR activity increases glucose and amino acid transporters on cell membrane, stimulates the hypoxia-inducible factor-α (HIF1-α), c-Myc, altogether maximizing nutrient uptake, glycolysis and glutaminolysis (88–90). Foxp3 suppresses c-Myc expression, thus limits glucose uptake and glycolysis of Tregs while promoting OXPHOS (91, 92). Hypoxia-driven HIF1-α activation has been shown to increase Treg fragility by binding Foxp3 and targeting it for proteosomal degradation (73, 93, 94). However, there is conflicting evidence on the overall effects of hypoxia on Tregs as, it also promotes TGF-β expression and signaling, hence can facilitate pTreg generation (95–97). In highly glycolytic tumors, glucose is broken down into lactate that can be taken up by Tregs *via* MCT1 lactic acid transporter. Tregs have been shown to metabolize lactic acid into phosphoenolpyruvate (PEP) in lactic acid rich, glucose poor environments. This has been shown to increase intracellular calcium and NFAT translocation in Tregs, but not in CD8^+^ T cells, and preserved suppressive abilities of Tregs *in vitro* (87). Mice with specific deletion of MCT1 lactic acid transporter in Treg compartment, displayed increased effector CD8^+^ and CD4^+^ T cell activity in the TME that was further enhanced upon PD-1 blockade (87). Tumor growth is also associated with increasing free fatty acid (FFA) content of TME (98–100). It has been shown that both tumor-associated myeloid cells and Tregs take up FFAs *via* scavenger receptor CD36 (101). CD36-mediated FFA signals through PPAR-β and promotes mitochondrial fitness and intratumoral Treg survival. Treg-specific deletion of CD36 selectively impairs intratumoral Tregs and reduces tumor burden without overt autoimmunity (102). Furthermore, comprehensive transcriptome analyses of Tregs from tumor and peripheral tissues of mouse melanoma, breast cancer and head and neck squamous cell carcinoma revealed that lipid metabolism is among the top enriched pathways among intratumoral Tregs (86, 103). This analysis also identified sterol-regulatory-element-binding proteins (SREBPs) as crucial transcription factors that control lipid metabolism and homeostasis of Tregs in the TME. Tregs deficient in SREBP1 cleavage activating protein (SCAP) were found less able to maintain a suppressive phenotype within the TME due to impaired SREBP activity (86, 104). Phenotype of Treg-specific SREBP1 deletion is similar to that of CD36, where mice don’t develop systemic inflammatory disorder, and have improved anti-tumor responses. It is worthwhile noting that SREBPs also play role in metabolism of healthy tissues, therefore broad targeting of SREBPs may pose serious risks for off-target toxicity (104). Lastly, Indoleamine 2,3-dioxygenase (IDO) is an enzyme expressed by APCs, epithelial cells, vascular endothelium and tumor cells that contributes to tolerogenic milieu in TME. It metabolizes the essential amino acid tryptophan into kynurenine, thus induces the stress-response kinase GCN2 that suppresses mTOR activity. Reduced mTOR activity can cause Foxp3 induction in effector T cells (105). Kynurenine has also been found to activate transcription of the aryl hydrocarbon receptor (AHR) that further promotes Treg differentiation. IDO is another attractive target for monotherapy and combination therapies with ICI (105–107).

## Targeting the suppression mechanisms used by Tregs in the TME

While reducing Treg infiltration of the TME is a desirable goal for cancer immunotherapy, targeting suppression mechanisms of Tregs may also provide good outcomes. This may especially be helpful in treating established tumors that have already been enriched with Tregs. Tregs have been shown to require an antigen encounter in the tumor-draining lymph node and subsequently within the tumor to optimally exert their suppressive functions (31). Activated Tregs can suppress APCs and effector T cells using both antigen-specific and bystander mechanisms. While their bystander effects are mostly mediated through suppressive cytokines, antigen-specific suppression relies on a contact-dependent removal of antigenic peptides (4). The use of these mechanisms by Tregs is dictated, for the most part, by antigen density and the inflammatory context it is presented in. Compared to a pathogen activated immune response, anti-tumoral immune response is surrounded by an immunosuppressive milieu that inhibits antigen presentation and overall lymphocyte function (108). In such a microenvironment, some Treg clones seem to proliferate and can possibly reach substantial numbers at later stages, outcompeting the effector T cells. Although reduced clonal diversity of Tregs may point out to a Treg response against particular tumor-derived antigens, it is hard to say that they operate in an antigen-specific manner (15, 40, 109). Instead, there may be unique combinations of antigen-specific and bystander mechanisms that Tregs tailor for the type and stage of the tumor based on the microenvironmental cues they receive. Although we are still a long way from deciphering the multiverse of suppression mechanisms, some useful clues from mouse models have started to point us in the right direction for finding Treg mechanisms that may be worth targeting.

### Contribution of Tregs to the regulatory milieu of TME

Tregs have been reported to release a plethora of cytokines that inhibit immune responses. Among those, IL-10, IL-35 and TGF-β have been intensely investigated for their contribution to Treg mediated suppression. While systemic effects of Treg specific deletion of these cytokines are controversial, their production by Tregs seem to be critical in particular niches such as epithelial barriers and TME (110–116).

IL-10 is a pleitropic cytokine that has immunoregulatory and immune activating effects. Treg-derived IL-10 has been shown to suppress antigen presentation and effector T cell function *in vitro*, and promote tolerance at the intestinal lamina propria *in vivo*. Although it is mostly produced by tumor associated myeloid cells and Tregs, overall effects IL-10 on tumor immunity remain elusive (117–119). It has been reported that tumor associated M2-type macrophages produce IL-10 to weaken the anti-tumor immunity in various cancer types (120, 121). Treg-derived IL-10 was shown to facilitate CD4^+^ and CD8^+^ T cell exhaustion by activating Prdm1 locus and upregulating BLIMP-1 mediated expression of PD-1, LAG3, TIM3, TIGIT, 2B4 (113, 122). On the other hand, studies that introduced PEGylated IL-10 for therapy elicited improved CD8^+^ TIL function and metabolic profile in mouse models and patients with solid tumors (123–127). Moreover, tumor targeted delivery of IL10-cetuximab fusion protein reduced apoptosis of intratumoral CD8^+^ T cells, boosted IFN-γ production and decreased tumor burden (124). A multicenter phase 1 clinical trial for PEGylated IL-10 monotherapy and combination therapy with anti-PD-1 also demonstrated expansion of antigen experienced CD8^+^ T cells in advanced tumor patients in both groups, further supporting an activating role for IL-10 in tumor immunity (128). However, the overall effect of Treg-derived IL-10 in TME and the strategies to target it require further elucidation.

IL-35 is a suppressive cytokine that belongs to IL-12 family. It is a heterodimer composed of Epstein-Barr-virus-induced gene (Ebi-3) and IL-12α chain (p35) and its expression has been detected in a variety of tumors (129, 130). A Treg specific Ebi3 deletion was shown to ameliorate anti-tumor responses similar to the effects observed in anti-IL35 treatment of wild type mice suggesting that depletion of IL-35 expressing Tregs and/or blocking IL-35 activity can be developed as potential immunotherapies (131, 132). IL-35 has been shown to act together with IL-10 in TME to induce BLIMP-1 mediated upregulation of inhibitory receptors on tumor-reactive CD4^+^ and CD8^+^ T cells (122). Furthermore, IL-35 release by Tregs has been shown to confer a regulatory phenotype to Foxp3- effector T cells in an IL-10 dependent manner (131). These cells, called iTr35, represent a stable suppressive population and can perform infectious tolerance in TME and transplant settings (133). More recently, IL-35 expression has been attributed a role in tumor cell extravasation and metastatic processes (134–136). Whether IL-35 producing Tregs are involved in metastasis remains elusive.

TGF-β is another cytokine that has important roles in immunoregulation and malignant transformation (137–139). High plasma TGF-β concentration and tumor TGF-β expression are used as indicators of poor prognosis in late stages of cancer and may help predict immunotherapy resistance in cancer patients (140–142). TGF-β blockade was shown to have a synergistic effect with checkpoint inhibitors in animal models, therefore, multiple clinical trials have investigated therapeutic effects of TGF-β inhibition as a monotherapy and in combination with ICIs (140). While TGF-β is secreted mostly by tumor and tumor associated fibroblasts, it can also be released by activated Tregs in a paracrine fashion. Upon activation, Tregs express the surface receptor glycoprotein-A repetitions predominant (GARP), which tethers inactive latent TGF-β complex to the Treg membrane. Upon cellular interactions, αVβ8 integrins can activate TGF-β in-cis and trans (143, 144). This provides a local, niche-restricted delivery of biologically active TGF-β by Tregs. Among T cells, GARP expression is limited to activated Tregs and in mice with Treg-specific GARP deletion, mice develop spontaneous colitis resembling the phenotype seen in Treg-specific TGF-β deletion. Furthermore, deletion of GARP in Tregs confer protection from colorectal cancer, suggesting that GARP may be an ideal drug target for inhibiting TGF-β mediated immunosuppressive effects of Tregs in colorectal tumors (145–149). GARP is also expressed by tumor cells, providing a reservoir of TGF-β, thus GARP inhibition may have broad tumor-directed effects beyond limiting Treg suppression (146, 150–152).

A potential mechanism whereby Tregs can alter TME is the removal of extracellular ATP by its surface ectonucleotidases, CD39 and CD73. Rising extracellular ATP largely reflects tissue injury and hypoxia and is perceived by the immune system as a danger signal (153). While low and moderate levels of extracellular ATP provide activating signals to T cells *via* purinergic P2XR receptors, high levels can be toxic leading to mitochondrial dysfunction and cell death. However, this is not a Treg-specific mechanism. In fact, a plethora of innate and adaptive immune cells, including myeloid cells, DCs, B cells, effector T cells and Tregs, can metabolize ATP *via* CD39 and CD73 (154). While CD39 degrades ATP into AMP, CD73 metabolizes AMP into immunoregulatory adenosine (155, 156). Adenosine binds to adenosine receptor 2a (A2a) on CD8+ T cells and NK cells and inhibit their anti-tumor activity (157, 158). Overall, targeting CD39, CD73 and/or A2a receptor one by one or in combination are attractive strategies to enhance anti-tumor immune responses.

### Costimulatory and coinhibitory pathways used by Tregs

**Figure 2:**
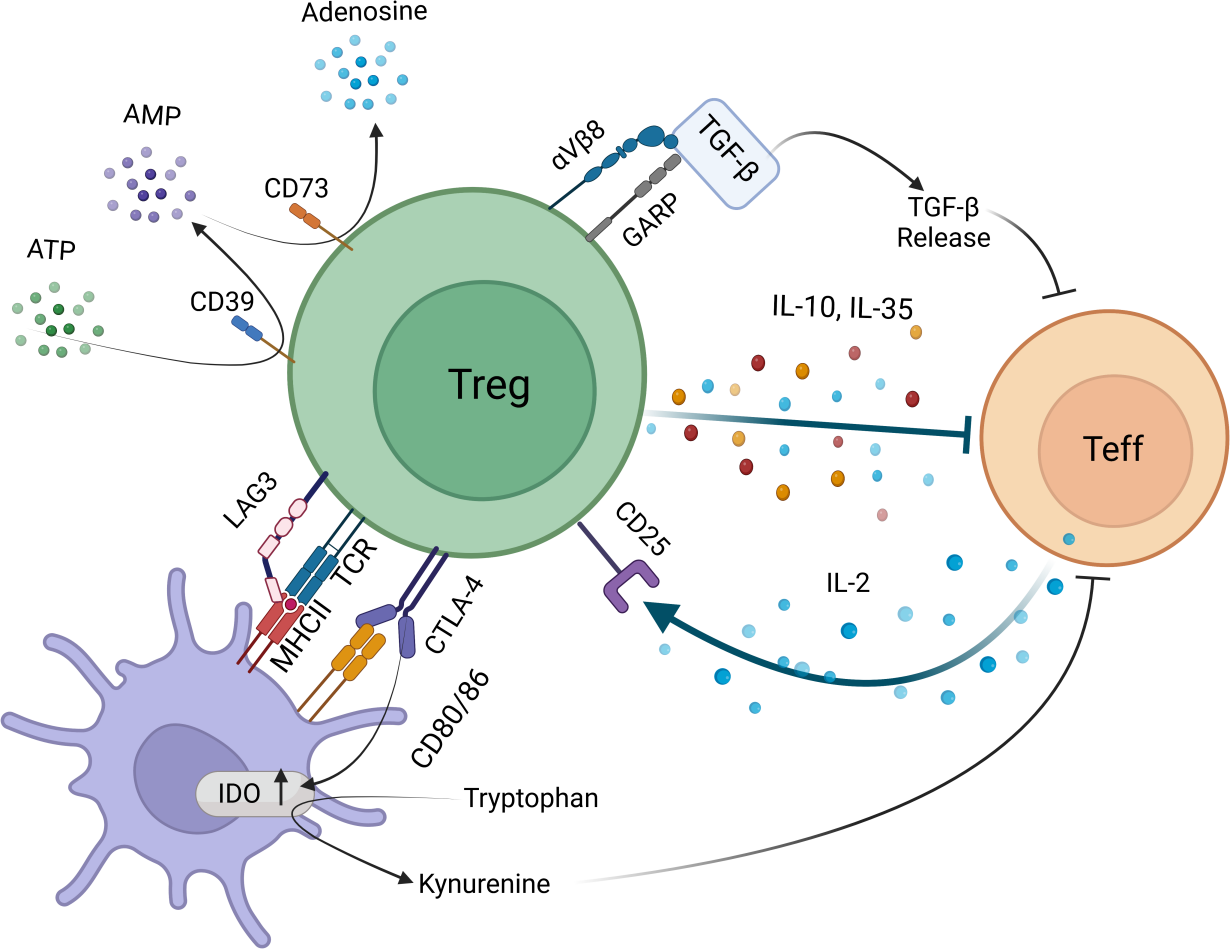
Targets for limiting intratumoral Treg activity based on in vivo mechanisms of suppression Treg-APC encounters activate antigen-specific and bystander suppression mechanisms. Tregs interfere with effector T cells function directly *via* producing inhibitory cytokines IL-10 and IL-35, delivering bioactive TGF-β *via* GARP-αVβ8 integrin axis, degrading proinflammatory ATP into AMP by surface 5’ ectonucleotidase CD39 and cleaving AMP into tolerogenic adenosine by 3’ ectonucleotidase CD73. Tregs can also deplete IL-2 by high affinity IL-2Rα (CD25), thus lead to effector T cell apoptosis. Indirect suppressive mechanisms include removal of antigen-MHCII and CD80-CD86 *via* TCR and CTLA-4 mediated transendocytosis and trogocytosis events. Tregs can also activate IDO activity *via* CTLA-4 reverse signaling into APC.

Tregs express a plethora of costimulatory and coinhibitory molecules, mainly members of the CD28-B7 family and the tumor necrosis factor receptor superfamily of receptors (159, 160). CTLA-4 is one of the most well-known coinhibitory receptors of CD28-B7 family that is constitutively expressed on Tregs while its expression on the effector CD4^+^ and CD8^+^ T cells is induced following activation (161). CTLA-4 regulates effector T-cell responses in cell intrinsic and extrinsic manners, cell-extrinsic functions primarily being operated by Tregs (162). CTLA-4 has been shown to quench the signals downstream of TCR and CD28 at the effector immune synapse, compete with costimulatory receptor CD28 for binding CD80-CD86, downregulate CD80-CD86 expression and remove CD80-CD86 from APCs *via* trogocytosis and transendocytosis (**Figure 2**) (7, 11, 162–167). However, whether Tregs use CTLA-4 as a major player in maintaining peripheral tolerance or suppressing anti-tumoral immunity is still debated. While a Treg specific deletion of CTLA-4 from birth causes severe autoimmunity and death, an induced deletion during adulthood displays no overt autoimmunity with a slightly enhanced resistance to experimental autoimmune encephalomyelitis (111). This discrepancy possibly points out the importance of CTLA-4 in Treg development instead of function, and also suggests a role for CTLA-4 in tuning the CD28-driven homeostatic Treg proliferation (111, 168). Furthermore, suppressive ability of CTLA-4 deleted Tregs were found unchanged, suggesting that CTLA-4 is not used as a standalone mechanism by Tregs (45, 111). However, the overall effect of Treg CTLA-4, if any, seems to be diminishing the duration and quality of the contacts between APCs and effector T cells within the TME (163). While the role played by CTLA-4 in the TME is still obscure, the depletion of Tregs *via* anti-CTLA-4 antibodies seems to be an efficient strategy to boost the anti-tumoral immune responses. Yet, due to the well-established role of CTLA-4 in maintaining peripheral tolerance, its broad inhibition poses a high risk for serious irAE (169). This highlights the need for developing more specific strategies and/or targeted drug delivery systems for anti-tumoral immunotherapeutics.

Similar to CTLA-4, PD-1 is also tasked to quench the activation signals transmitted by TCR and CD28 and plays a central role in the immunosuppressive nature of the TME (170). High PD-1 expression is one of the hallmarks of exhausted phenotype in T cells, hence the blockade of PD-1-PD-L1 axis has been adapted as another ICI modality (171, 172). Inhibiting PD-1 activity using antibodies against PD-1 or PD-L1 has been shown to rejuvenate exhausted effector T cells and reduce tumor burden (169, 171). While this therapy has already made its ways to clinics, our understanding of its action is largely limited to CD8^+^ T cells. However, a number of recent studies attempted to lay out role of PD-1 in Treg biology and maintenance in the TME (170). For instance, mice with Treg specific deletion of PD-1 displayed an activated phenotype and their increased suppressive abilities ameliorated autoimmune pathology (173). In the TME, high PD-1 expression on Tregs seems to be associated with reduced suppressive ability, therefore, blocking PD-1 pathway may revive those Tregs helping them more effectively compete with the effector T cells (174). Recent findings demonstrated that PD-1 blockade is less effective in tumors that are enriched with PD-1^high^ Tregs, supporting the risks associated with activating Tregs in the TME (171, 175, 176). This may also explain why ICI is more effective when it is combined with Treg-depleting strategies. The synergistic effect of anti-CTLA-4 (Ipilimumab) and anti-PD-1 (nivolumab) may also point out to the benefit of removing Tregs to make ICI more effective (177). However, clinicians should carefully evaluate the options and timing for monotherapy vs combination therapies case by case to keep irAE at bay (177, 178). In the context of new combination therapies, dual PD-1 and LAG3 inhibition has gained attention to reverse T cell exhaustion (179, 180). Similar to PD-1, LAG3 expression also correlates with CD8^+^ T cell dysfunction that is characterized by reduced capacity for proliferation and cytokine production (181–183). As a corollary, LAG3 blockade reinvigorates CD8^+^ T cell function however, its role in CD4^+^ effector T cells is rather complex. Extracellular portion of LAG3 is cleaved off by metalloprotease enzymes ADAM10 and 17 upon activation and released as soluble monomeric LAG3 (sLAG3). LAG3 shedding was found critical for CD4^+^ T cell function in an elegant mouse model where CD4+ T cells were introduced a non-cleavable LAG3 mutant (184). This may be due to an incessant inhibitory signal through mutant LAG3 which would otherwise stop because of shedding. Alternatively, sLAG3 shed by CD4^+^ T cells has a local stimulating effect on CD4^+^ T cells or DCs in an autocrine or paracrine fashion. LAG3 harbors further complexities in Treg compartment. A Treg-specific deletion of LAG3 from birth was found to reduce autoimmune diabetes incidence and severity in NOD mice and diminish immune infiltration of pancreas (185). This was possibly due to exhausting effect of LAG3 expression on Tregs, similar to that of PD-1 due to chronic stimulation within tissue (175, 176). On the other hand, in experimental autoimmune encephalomyelitis and adoptive transfer colitis models, adoptive transfer of LAG3 deficient Tregs failed to protect against disease despite normal trafficking and stable foxp3 expression suggesting that LAG3 can be required for Treg mediated suppression in certain autoimmune settings while it can be detrimental in others (186, 187). However, we still don’t know which category tumor-specific Tregs fall in the spectrum of LAG3 mediated effects.

Another costimulatory molecule that plays role in fine-tuning Treg activity is GITR (188). GITR is a member of the TNFR superfamily of coreceptors that is constitutively expressed at high levels on Tregs (189). Naïve effector T cells also harbor a low surface expression that is upregulated upon activation (190). GITR seems to play a complex, context-dependent role in the immune system and so far, it has been shown to promote effector T cell activity in a cell-intrinsic manner, induce Treg expansion and inhibit Treg suppressive function (160, 188, 191, 192). In the context of tumors, stimulation of GITR pathway has been shown to destabilize Tregs, equipping them with cytotoxic abilities (192). While the effect of GITR monotherapy appears to be limited and varies with the tumor type, adding GITR agonists to PD-1 blockade has been shown to potentiate its anti-tumor effect (160, 192). Therefore, GITR agonism presents a promising new strategy for cancer immunotherapy hence, there are multiple ongoing clinical trials investigating the effects of GITR agonism and its combination with ICI in different cancer types (193, 194).

### Strategies harnessing TCR and CD25 for eliminating tumor-specific Tregs

CD25 is the high affinity IL-2 receptor expressed constitutively at a high level by Tregs. It also gets expressed on effector T cells, B cells, NK cells upon activation (195–197). While a constitutive high expression seems to make it a reliable marker for murine and human Tregs, CD25 is not enough on its own and needs to be combined with other indicators such as Foxp3, Helios and/or lack or low expression of CD127 to determine Treg lineage (198). Likewise, failure of CD25 in distinguishing Tregs and activated effector T cells at sites with an ongoing immune response, such as the TME, makes it an unreliable target for immunotherapy. Indeed, CD25-targeting agents can hamper the anti-tumor immunity by depleting activated effectors or restricting their access to IL-2 and this may explain, at least partially, why anti-CD25 antibodies that have been used to deplete Tregs from the TME have not yielded optimal results (196, 197, 199, 200). Another possible reason could be the selection of an anti-CD25 antibody that suboptimally engages the Fc receptors in the TME, leading to a reduced ADCC (196). These setbacks have led to an effort to develop antibodies with better characteristics such as an improved intratumoral ADCC capability and an incessant IL-2 signaling in the effector T cells. These strategies, while not completely specific for Tregs, have been shown to be effective in boosting anti-tumor T cell response and reducing tumor burden in mice (197). Furthermore, their combination with PD-1 inhibitors has demonstrated significant synergistic effect, somewhat similar to the combination approaches using CTLA-4 (196). Yet, these new approaches must be carefully tested in clinical trials as the evidence for their ability to discriminate peripheral vs. intratumoral Tregs in human body are still lacking. This is a key safety point that should not be overlooked as the attempts to elicit better Treg depletion strategies may lead to intractable irAE.

**Figure 3:**
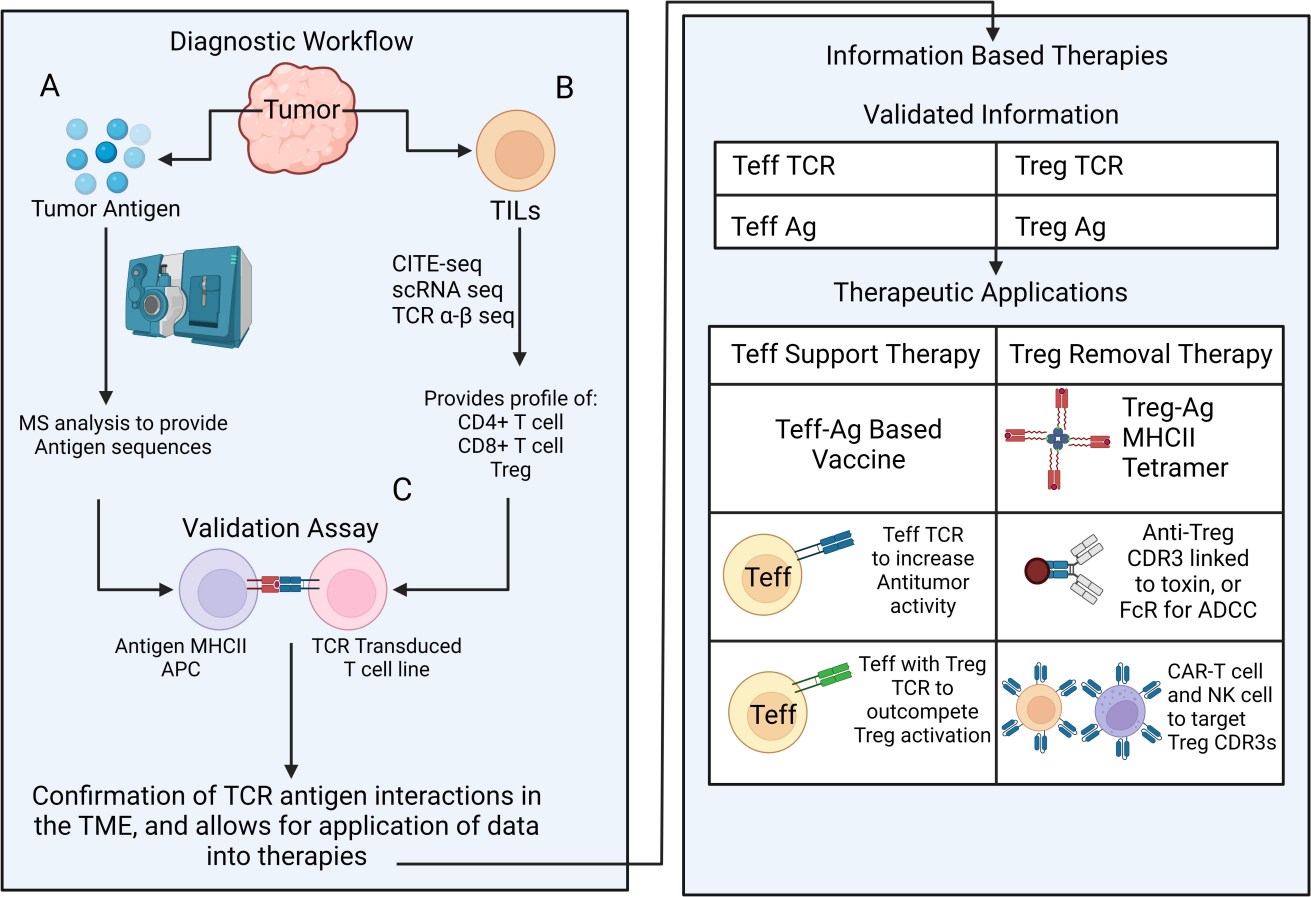
A multipronged approach for outcompeting vs. eliminating tumor-specific Tregs Diagnostic workflow for revealing antigen-specific elements in tumor tissue includes a multi-pronged approach; **(A)** Mass spectrometry-based detection of candidate antigens that potentially drive effector or regulatory T cell expansion, **(B)** Single cell RNA and TCR αβ VDJ sequencing for identifying and phenotyping the most frequently represented CD4+, CD8+ and Treg clones. **(C)** Next step aims at selecting biologically relevant antigen-TCR combinations *in vitro*. Validated antigen and TCR information can be used to develop strategies to selectively boost effector T cells and/or eliminate Tregs. Strategies for effector T cell support (Teff support therapy) include antigen vaccines, adoptive transfer of TCR transgenic effector T cells that contain select effector T or Treg TCRs. Strategies for Treg inactivation/removal consist of Treg antigen (ag) -MHCII tetramer to cease Treg-APC contacts, antibodies targeting select Treg TCR-CDR3 for removal (conjugated to a toxin or *via* FcR mediated ADCC) and CAR-T or CAR-NK cells targeting Treg TCR-CDR3 for Treg removal.

One poorly understood characteristic of the TME that future therapies can focus is the oligoclonal, potentially tumor-specific, expansion of the intratumoral Tregs. As was shown for secondary lymphoid organs *in vivo*, it is possible that the TME harbors a competition between the effector T and Treg clones for the tumor neoantigens (45). If so, a slow take over by the neoantigen-specific Tregs may gradually transform the clonal landscape of the TME for the intratumoral neoantigen-specific T cells *via* mechanisms such as antigen-specific suppression by pMHCII depletion and/or conversion into tumor-specific pTregs due to suboptimal antigen stimulus and elevated TGF-β as well as bystander mechanisms of suppression (40, 109). Unfortunately, it has so far been difficult to capture these possibilities due to a lack of tools such as reliable markers to distinguish tTreg and pTregs and time-sensitive sampling and analysis for the clonal representation and antigen-specificity of Tregs and effector T cells. Until we have a better understanding for how bystander and antigen-specific suppression mechanisms operate in the TME, we can utilize TCR sequence and antigen specificity of intratumoral Treg clones as a proxy to understand the type of response that needs to be potentiated (**Figure 3**). This would facilitate new antigen-targeted cellular biotherapies such as TCR-transgenic T cell, CAR-T cell and antigen-loaded immunogenic DCs and would ultimately revive or deliver right set of effectors that can outcompete intratumoral Tregs.

### Conclusion

The fine balance between intratumoral Tregs and effector T cells is closely linked to disease prognosis. Tregs have a myriad of adaptations that facilitate their survival and function in TME. They can metabolize FFAs and lactic acid, hence survive in low glucose, low oxygen environments. Tolerogenic milieu enriched in TGF-β, adenosine and kynurenine can also induce Foxp3 expression in effector CD4^+^ T cells, further contributing to intratumoral Treg pool. Several contact-dependent and paracrine mechanisms have been proposed for Treg mediated suppression. However, strategies targeting Tregs have so far been hampered by our limited success in delineating how they suppress in-situ. This has been compounded by a lack of reliable markers that discern tumor-specific Tregs from tumor-specific effector CD4^+^ T cells and self-reactive Tregs, taking us back to the drawing board due to unpredictable risks of harming peripheral tolerance. Although antigen-targeted precision therapies have a long way from clinics, they appear to be a safer route to limit immune related adverse events. By unraveling immune interactions taking place in TME thoroughly, we would be able to lend a hand to the right effectors in their tug of war with intratumoral Tregs.

## Author contributions

BA conceived the manuscript. BRM and BA drafted the manuscript. BA performed the critical revision and finalization of the manuscript. All authors contributed to the article and approved the submitted version.

## Funding

This work was supported by the National Institutes of Health grant number DP2AI154451.

## Conflict of interest

The authors declare that the research was conducted in the absence of any commercial or financial relationships that could be construed as a potential conflict of interest.

The handling editor declared a shared affiliation with the authors at the time of the review.

## Publisher’s note

All claims expressed in this article are solely those of the authors and do not necessarily represent those of their affiliated organizations, or those of the publisher, the editors and the reviewers. Any product that may be evaluated in this article, or claim that may be made by its manufacturer, is not guaranteed or endorsed by the publisher.
